# *Notes from the Field:* An Outbreak of Shiga Toxin–Producing *Escherichia coli* O157:H7 Associated with a Farming Camp — Tennessee, 2022

**DOI:** 10.15585/mmwr.mm7229a6

**Published:** 2023-07-21

**Authors:** Lindsey Ferraro, D. J. Irving, Jack Marr, Kelly Orejuela, Erin Murray, Mugdha Golwalkar, Lisa M. Durso, Julie Viruez, Robin Rasnic, Katie Garman, John Dunn

**Affiliations:** ^1^Tennessee Department of Health; ^2^Agricultural Research Service, U.S. Department of Agriculture, Lincoln, Nebraska; ^3^Division of Laboratory Services, Tennessee Department of Health, Nashville, Tennessee.

On June 22, 2022, the Tennessee Department of Health (TDH) was notified of a child hospitalized with Shiga toxin–producing *Escherichia coli* (STEC) O157:H7 after attending a farming camp at farm A. Three days later, TDH was notified of a second hospitalized child with hemolytic uremic syndrome, whose brother had attended the same camp, prompting an investigation. During the summer, farm A held three week-long summer camps teaching animal husbandry to children aged 6–10 years by assigning campers a baby goat (kid) to care for. STEC resides in the gastrointestinal tract of ruminants such as cattle, goats, sheep, and deer without causing illness in the animal* (*1*). Outbreaks among humans associated with petting zoos are well documented (*2*–*5*).

## Investigation and Outcomes

On June 28 and 29, TDH conducted an environmental assessment at farm A. In addition to an onsite interview with the farm owners and employees, the assessment included facility observations of animal pens, public petting areas, areas where children cared for the animals, food service facilities, handwashing and sanitizing facilities, play areas, and toilets. Health department staff members collected camp attendee registration and goat assignment records and conducted environmental sampling, including the collection of 41 samples from animals, animal feces, animal pens, water sources, and toilets.

TDH also sent an online survey to the parents and guardians of all 82 children who had attended camp at farm A during June 6–24 to ascertain dates of attendance, illnesses and outcomes, foods consumed, and camp activities. The outbreak-specific survey was completed by parents or guardians of 53 (65%) campers.

Survey responses facilitated conduct of a case-control analysis. Cases were defined in terms of 1) the person who was ill (primary versus secondary) and 2) the symptoms and laboratory results (probable versus confirmed). A primary case was defined as an illness in a person who attended any of the three camps during June 6–24; a secondary case was a compatible illness within 10 days of exposure to a primary case in the same household or to a close contact of a summer camp attendee (irrespective of illness in the attendee). Probable cases included the onset of diarrhea within 10 days of attending the summer camp (primary cases) or within 10 days of exposure to a secondary case; confirmed outbreak cases were defined as a positive polymerase chain reaction or enzyme immunoassay Shiga toxin test result from a specimen collected after June 6.^†^ Twelve primary cases (including two confirmed and 10 probable) and two secondary cases (one confirmed and one probable) were identified (patient age range = 2–38 years) (Figure). One patient each with a primary and secondary case was hospitalized; one death occurred in a child aged 2 years with a secondary confirmed case.

**FIGURE:**
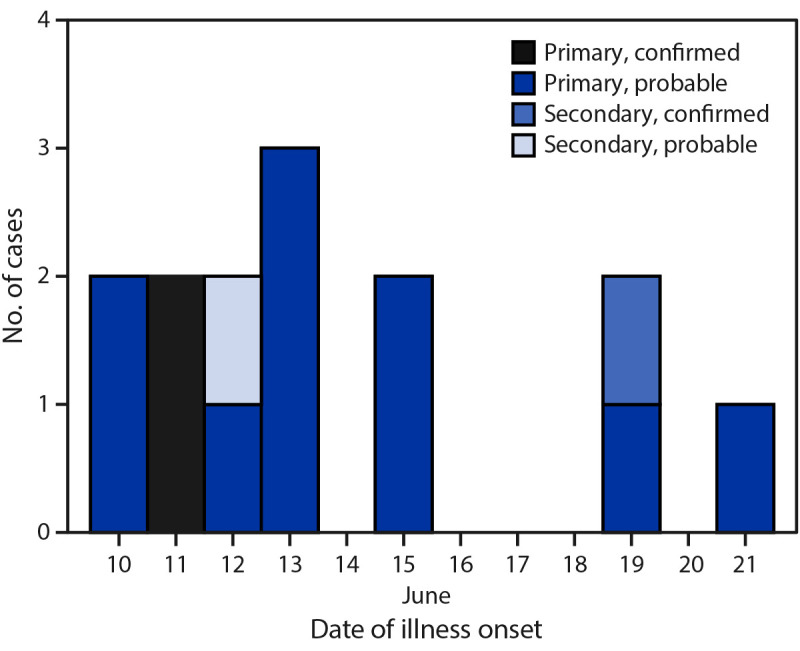
Onset of primary,* secondary,^†^ probable,^§^ and confirmed^¶^ cases of Shiga toxin–producing *Escherichia coli* O157:H7 illness among persons associated with a farming camp (N = 14) — Tennessee, June 2022 * An illness in a child who attended any of the three farm A summer camps during June 6–24, 2022. ^†^ A compatible illness in a household member or close contact of a farm A summer camp attendee. ^§^ The onset of diarrhea within 10 days of attending the farm A summer camp (primary cases) or within 10 days of exposure to a patient with a primary case (secondary case). ^¶^ A positive stool culture test result for Shiga toxin–producing *Escherichia coli* from a specimen collected after June 6.

The case-control analysis included 12 ill camp attendees as case-patients and 58 healthy children identified from the camp attendee list as controls. Chi-square analysis was used to calculate odds ratios; 95% CIs that excluded 1 were considered statistically significant. Because the camp’s food and activity schedules did not change between weeks, and no contributing factors were identified in farm A’s food service establishment, neither a specific activity nor food was considered to be associated with illness. Attendance during the first week of camp, however, was significantly associated with illness (odds ratio = 13.1; 95% CI = 2.59–66.57). Camp operators reported being aware of the National Association of State Public Health Veterinarians Animal Contact Compendium^§^ and reported incorporating handwashing stations, observing children during animal interactions, and keeping the animal areas clean and disinfected.

Investigators were able to isolate STEC by culture in six samples collected at farm A; these were further subtyped into three STEC serotypes by core genome multilocus sequence typing: H14 (one rectal swab [kid] and one stool swab [kid]), O157:H7 (one stool swab [kid] and one wood swab [inside kid barn]), and O26:H11 (two stool samples [kids]).^¶^ Only STEC O157:H7 was associated with clinical illnesses. The two farm A STEC O157:H7 isolates were closely related by whole genome sequencing to the three outbreak-associated STEC O157:H7 patient isolates.

## Preliminary Conclusions and Actions

In response to the outbreak, farm A voluntarily closed the camp, expedited the demolition of the kid barn, euthanized two kids with positive STEC test results, and moved the kid herd off the property. During closure, farm A independently consulted with veterinarians and other petting zoos to identify additional methods for reducing disease transmission. Based on recommendations provided, the facility discontinued the animal husbandry portion of the camp, increased signage encouraging handwashing after touching animals or objects throughout the facility, and increased messaging on their website about zoonotic diseases, populations at highest risk, and ways to mitigate risk for infection. On July 18, farm A reopened their summer camp without the goat husbandry component.

TDH concluded that this outbreak was associated with STEC O157:H7-infected kids and involved secondary transmission. Hand-to-mouth contact has been observed to occur almost three times per hour among children aged 6–10 years,** supporting the potential for STEC ingestion from contaminated environmental surfaces. The hypothesis of prolonged contact between campers and kids resulting in illness is strengthened by the finding that, after conducting routine monitoring of pathogen and case report forms as well as complaint surveillance systems, STEC was not identified by patrons of the farm apart from camp attendees and their household members. Animal farms, petting zoos, and other environments where small children might have direct contact with ruminant animals should be aware of the risk for zoonotic STEC transmission and make efforts to mitigate these risks by promoting proper hand hygiene during and after animal contact.

## Footnotes

*
https://www.cdc.gov/healthypets/diseases/ecoli.html

† All persons who received testing were tested by their health care provider. Providers sent two positive specimens to the Tennessee State Public Health Laboratory for sequencing, and the third was sequenced by the Florida State Public Health Laboratory because the child became ill and was hospitalized in Florida.

§
http://nasphv.org/documentsCompendiumAnimals.html

¶ The remaining 35 samples tested negative, including one swab each from three goats, four from well water (mother goat drinking water, kid drinking water, pond, and water pipe), four from treated well water, eight from the barn environment (including one from goat feed), three from portable restrooms, 10 goat feces specimens, two lamb feces specimens, and one stool specimen from an unknown animal source.

**
https://onlinelibrary.wiley.com/doi/10.1111/j.1539-6924.2007.00893.x
